# Economic Evaluation of a Novel Physical Healthcare Advice and Guidance Service in a Mental Health Trust

**DOI:** 10.1192/bjo.2025.10088

**Published:** 2025-06-20

**Authors:** Eoin Gogarty, Ge Yu, Ioannis Bakolis, Ray McGrath, Fiona Gaughran

**Affiliations:** ^1^Psychosis Studies, Institute of Psychiatry, Psychology and Neuroscience, King’s College London, London, United Kingdom; ^2^NIHR Applied Research Collaboration South London, London, United Kingdom; ^3^King’s Health Economics, Health Services and Population Research, Institute of Psychiatry, Psychology & Neuroscience, King’s College London, London, United Kingdom; ^4^Centre for Implementation Science, Health Service and Population Research Department, Institute of Psychiatry, Psychology and Neuroscience, King’s College London, London, United Kingdom; ^5^Department of Biostatistics and Health Informatics, Institute of Psychiatry, Psychology and Neuroscience, King’s College London, London, United Kingdom; ^6^South London and Maudsley NHS Foundation Trust, London, United Kingdom; ^7^King’s Health Partners, Mind and Body Programme, Ground Floor, Counting House, Guy’s Hospital St Thomas St, London, United Kingdom; ^8^National Psychosis Service, South London and Maudsley NHS Foundation Trust, London, United Kingdom

## Abstract

**Aims:** People with severe mental illnesses experience poorer physical health outcomes compared with the general population, partially related to fragmented care. The Integrating our Mental and Physical Healthcare Systems project implemented an Advice and Guidance Line, supported by colleagues in King’s Health Partners, using the Consultant Connect (CC) app in the South London and Maudsley NHS Foundation Trust to enhance collaborative physical healthcare. This study evaluates the app’s impact on inpatient transfers from mental health wards to acute hospitals, focusing on clinical outcomes and cost savings.

**Methods:** This cost-minimisation analysis used retrospective observational data to analyse electronic health records across a 42-month period (21 months pre- and post-intervention) centred on the CC introduction date in June 2020. The study population was Trust adult inpatients during the study period. Outcome measures were the number of Trust inpatients who attended ED in, or were admitted to, one of the four acute NHS Trusts serving the catchment area. Transfers with a primary COVID-19 diagnosis were excluded. Outcomes are presented as the number of transfers per Trust inpatient bed-year. This divisor accounts for the decrease in bed-days during the pandemic.

**Results:** In the pre-CC period there were 5,472 Trust inpatients across 7,308 inpatient episodes (1,328.78 bed-years) with 1,834 ED transfers. Post-CC the Trust had 5,362 inpatients across 7,396 episodes (1,183.06 bed-years) with 530 ED transfers. The number of ED transfers per bed-year was 1.38 in the pre-CC period, and 0.45 in the post-CC period, a 68% reduction (p<0.001, Chi-square). Interrupted time-series analysis confirmed this decrease (−0.752, 95%CI [−1.117, −0.386], p<0.001). There was no significant difference in admission rates pre- and post-intervention. Based on recent annual bed occupancy (720.97 bed-years) and costs (£457 per ED transfer), CC prevents approximately 670 transfers annually, generating total Trust savings of £241,720 after deducting annual service costs (£61,698) and annualised implementation costs (£3,000).

**Conclusion:** While the pandemic contributed to an initial decrease in ED transfers, the reduction was sustained even as overall ED presentations at the four hospitals returned to pre-pandemic levels. There was no change in admissions to acute Trusts, suggesting that the level of care provided was appropriate to need. The Advice and Guidance model appears cost-effective in managing physical health within mental health settings. These findings support wider implementation of similar services across mental health trusts, though further evaluation in a post-pandemic context is warranted.

